# Schizophrenia: temporal imprecision in phase, time, and frequency domains

**DOI:** 10.1038/s41537-026-00792-5

**Published:** 2026-08-12

**Authors:** Saeid Kholghi, Georg Northoff

**Affiliations:** Institute of Mental Health Research, University of Ottawa, Ottawa, ON Canada

**Keywords:** Neural circuits, Biomarkers

## Abstract

Schizophrenia is associated with deficits in input processing, but the underlying neural dynamics of this deficit remain unclear. A recent model proposes temporal imprecision as a basic disturbance driving the input processing deficit. To address the question of whether temporal imprecision affects all three domains of the brain’s signal (e.g., phase, time, and frequency) as early as first episode schizophrenia, we analyzed magnetoencephalography (MEG) data from 25 first-episode schizophrenia (FESZ) participants and 24 healthy controls (HC) during a passive auditory oddball task with standard, pitch deviant, and duration deviant tones while watching a silent movie. We quantified temporal precision via (i) intertrial phase coherence (ITPC), (ii) intrinsic neural timescale (INT), and (iii) power spectral density (PSD) heterogeneity. For all three signal domains, we also calculated their temporal variability in sliding windows over the MEG recording. FESZ showed reduced intertrial phase coherence to both deviant tones, with a broader time and frequency range for the duration deviant ITPC reduction. Intrinsic neural timescales were prolonged in FESZ subjects. PSD profiles were more heterogeneous and broader during the auditory task in the FESZ group. FESZ subjects also showed increased temporal variability in all three signal domains across sliding windows. Finally, significant associations were observed between temporal variability in all three signal domains, including their association to impaired duration deviant mismatch negativity amplitude in first episode schizophrenia, whereas these associations were largely absent for healthy participants. We conclude that first-episode schizophrenia shows convergent temporal imprecision at specific time points as well as between time points in all three signal domains including phase, time, and frequency; this is consistent with temporal imprecision as a domain-general basic disturbance of input processing in schizophrenia.

## Introduction

Schizophrenia (SZ) is a complex disorder with a yet unclear etiology that has been explored and studied from a variety of lenses from molecular to behavioral level. Studies using brain imaging technologies such as electro/magnetoencephalography (EEG/MEG) and functional magnetic resonance imaging (fMRI) have explored the systemic activity of the brain in schizophrenia patients in resting state and in response to different stimuli or tasks. Summarizing, these results show that SZ participants exhibit a general deficit in input processing, that is, across the different sensory modalities and cognitive domains^1–4^. The exact neural correlates of such basic input processing deficit, that is, its underlying neural dynamics, remain yet unclear, though. Addressing this gap in our knowledge is the goal of our paper.

One recent pathophysiological model of SZ proposes temporal imprecision in milliseconds as a key disturbance^1,5,6^. One established evidence of this deficit is the impaired temporal precision of the brain’s neuronal activity in tuning itself to an external repetitive auditory tone, like in oddball, mismatch, and steady-state paradigms^1,5–7^. This process is called entrainment^8^, and can be measured by intertrial phase coherence or clustering (ITPC). ITPC measures similarity or dissimilarity of the phase angle (where the signal is in its cycle) in the brain signal across different trials^9^; if the neuronal activity is not consistent in tuning to the precise timing of the stimuli across trials, it will have a different phase angle at each trial with high phase angle variance, resulting in low ITPC. In contrast, if the timing is consistent, phase angles over trials will be similar, hence yielding low phase angle variance with high ITPC. Taken in this way, high levels of ITPC are a measure of temporal precision, whereas low ITPC indicates temporal imprecision; the latter has often been observed in SZ^1,6,10^. How such decreased phase-based ITPC in SZ relates to other neuro-dynamic measures of the signal’s temporal precision in both the time and frequency domains during input processing remains unclear, though.

Input processing is not limited to only affecting the brain’s phase angles and their timing. Going beyond phase dynamics, the brain also exhibits so-called intrinsic neural timescales (INTs). INTs refer to temporal windows of self-similarity in the brain signal over time^11–13^, which, for instance, can be measured by the autocorrelation window (ACW)^14–19^. The brain uses its repertoire of different INTs to process and sort through incoming inputs and stimuli of different timescales, which allow for the temporal integration and segregation of distinct inputs^11–15,17–19^. Recent studies in SZ show abnormally long INT during both rest and task states in EEG/MEG, thus suggesting temporal imprecision in the time domain of INT during input processing^10,20^. However, few studies have investigated both INT and ITPC in the same schizophrenia subjects.

Besides phase dynamics and the time domain with INTs, another important dynamic feature of the brain’s signal is its frequency domain; this can, for instance, be measured by power spectral density (PSD), which shows the power of different frequencies from slow to fast in the brain’s signal. Previous studies have reported abnormal PSD characteristics in schizophrenia, marking an input processing deficit in the frequency domain^21–24^. This leaves unclear whether such PSD deficit during input processing is due to temporal imprecision in its periodic-oscillatory or aperiodic-fractal activity within the power spectrum^22^. Moreover, it remains unclear whether such PSD deficit co-occurs with corresponding changes in INT and ITPC in schizophrenia—in that case, one would assume that the temporal imprecision of input processing is also manifest in the frequency domain of the PSD. Given the potential occurrence of temporal imprecision in all three basic components of the neural EEG/MEG signal, namely phase dynamics (ITPC), time-domain (INT), and frequency domain (PSD), one may be inclined to characterize temporal imprecision as a basic disturbance of SZ^25^.

### Aims and hypothesis–temporal imprecision as basic disturbance of Input processing in schizophrenia

**Fig. 1:**
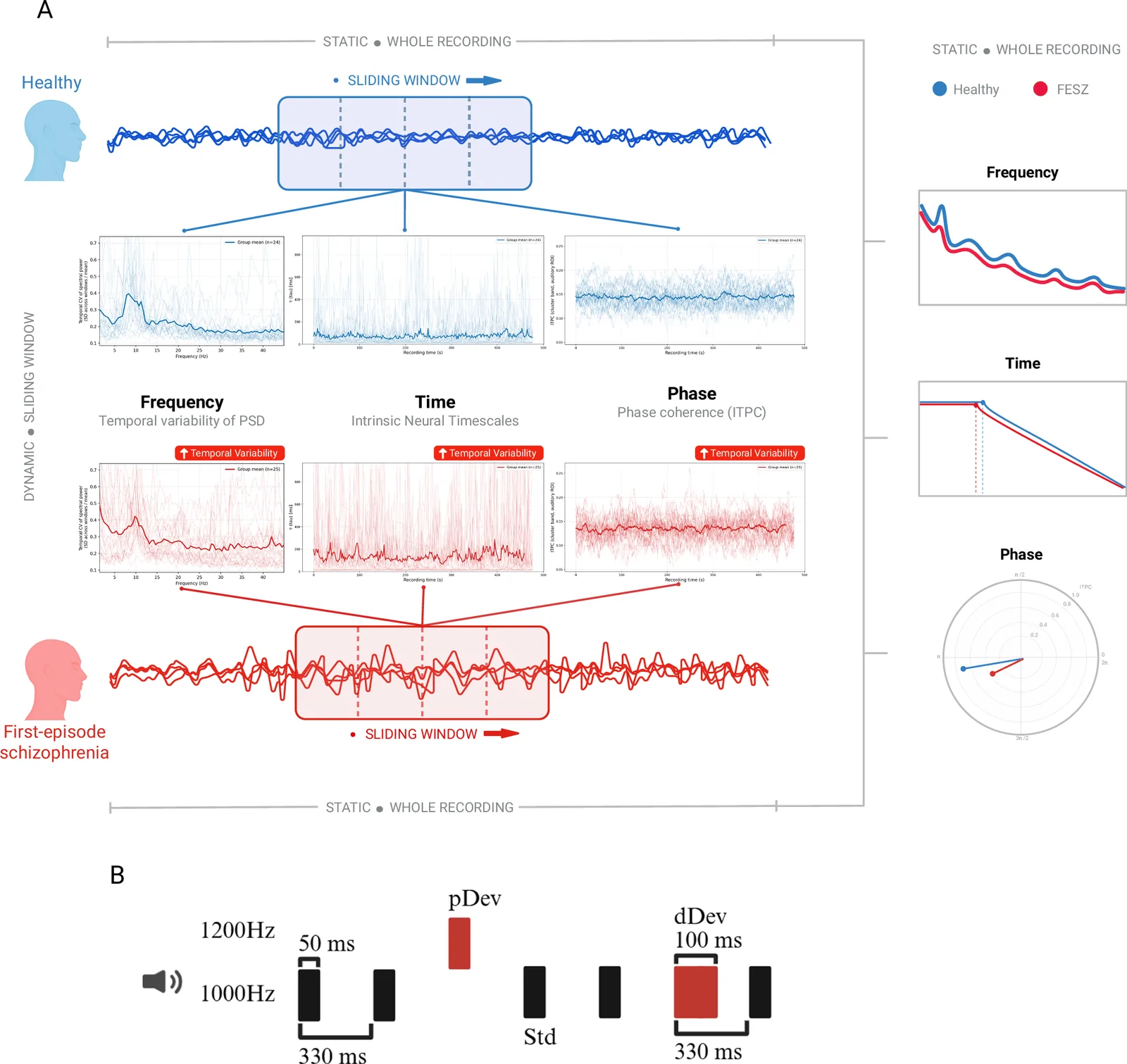
Temporal imprecision model and the auditory oddball paradigm. **A** Temporal imprecision in phase, time, and frequency domains. The dynamic panels from left to right correspond to, spectral coefficient of variation across sliding windows plotted against frequency, intrinsic neural timescales, and phase coherence across sliding windows. In each plot, faint graphs correspond to individual subjects and the bold graph to the group average. First episode schizophrenia shows increased temporal variability in all three domains. Static (average across the entire recording) results show more heterogeneous power spectra in first episode patients, resulting in less defined peaks, prolonged intrinsic neural timescales measured by the aperiodic knee of the power spectrum and reduced intertrial phase coherence. Static plots are data inspired schematics. **B** Standard tones (Std; 1000 Hz, 50 ms) were presented with a 330 ms stimulus-onset asynchrony and occurred on 80% of trials. Pitch deviants (pDev; 1200 Hz, 50 ms) and duration deviants (dDev; 1000 Hz, 100 ms) each occurred on 10% of trials. This figure was created in BioRender under the agreement number IZ2A1IACXC.

The present study addresses the question of whether temporal imprecision in schizophrenia is manifest in all three key domains of the brain’s signal, e.g., phase (ITPC), time (INT), and frequency (PSD) domains in an early stage of the disorder; in that case, temporal imprecision may be considered a basic disturbance^1,10,25^. (Figure 1A) We used magnetoencephalography (MEG) data recorded from 33 first-episode psychosis (FEP) patients and 24 healthy controls (HC). All individuals in the FEP group received their diagnosis within less than a year of recording the data.

Within this cohort, 8 patients have received diagnosis of affective disorders with psychotic features (FEAFF), and the remaining 25 participants a diagnosis of first-episode schizophrenia spectrum (FESZ). For our analysis, we focused on the FESZ subgroup.

The auditory paradigm presents three tone types: standard (regular), pitch-deviant (frequency change relative to standard), and duration-deviant (duration change relative to standard) (Fig. 1B). Given that the dataset follows a mismatch negativity design, we also calculated mismatch negativity (MMN) to confirm previous results^26^. Going beyond the amplitude of the MMN, this design enables a targeted test of temporal precision of the three key signal domains, phase, time, and frequency, in input processing, especially by testing the effects of temporal precision of the input duration, e.g., duration-deviant. For that purpose, our first aim was to test for temporal imprecision in the phase domain by measuring ITPC during the different conditions; we expected strongly decreased ITPC in FESZ during especially the duration-deviant as that tests for how the brain’s processes deviations in stimulus timing or duration. This would show direct relation of temporal imprecision in the input with the brain’s own temporal imprecision in the phase domain of the signal. Beyond the intertrial phase coherence for different tones across the entire recording, we also calculated phase coherence in a dynamic way, that is, dynamic ITPC (dITPC), to test whether first episode schizophrenia patients also exhibit increased temporal variability of ITPC across sliding windows, as it is the case for later stages of the disorder^6^.

The second aim was to investigate temporal imprecision in the time domain. For instance, temporal precision can also be operationalized through variability: higher variability implies lower temporal precision, whereas lower variability implies higher temporal precision. Following this logic, our second aim is to quantify within-subject (intrasubject) variability of INTs over the recording session, and to test whether the variability of INT differs between groups. Given our background model of temporal imprecision, we hypothesized that FESZ would show higher variability of INT compared to healthy subjects—this suggests higher temporal imprecision in the time course of the INT, and thus in the time domain during input processing.

Finally, our third aim was to test temporal imprecision also in the frequency domain of the PSD, including the separation of its periodic-oscillatory and aperiodic-fractal components^27^. We hypothesized that SZ subjects can no longer track and follow the input dynamics with their own intrinsic frequencies, that is, the various frequencies of their brain’s PSD, resulting in a more heterogeneous spectrum profile.

## Materials and methods

### Participants

Magnetoencephalography (MEG) data were taken from the Pittsburgh Early Psychosis Program (PEPP) mismatch-negativity^26^. The original sample comprised 26 healthy controls and 33 first-episode psychosis (FEP) participants recruited from UPMC Western Psychiatric Hospital inpatient and outpatient services. After preprocessing and artifact rejection, 24 controls (Age: 25.2 ± 5.1 years (range: 18.5–37.0, Sex: 13 male (54.2%), 11 female (45.8%)) and 25 FESZ participants (Age: 22.7 ± 3.9 years (range: 18.4–34.5), Sex: 19 male (76%), 6 female (24%)) remained for the present analyses.

FESZ participants met DSM-IV criteria for schizophrenia spectrum. At the time of MEG recording, 23 of the 25 FESZ participants were receiving antipsychotic medication, whereas two were unmedicated. Among medicated participants, the mean chlorpromazine-equivalent dose was 262.5 ± 167.6, and the mean interval between antipsychotic treatment initiation and MEG recording was 68.5 ± 90.6 days. Information regarding specific antipsychotic medications was not available in the secondary dataset. All participants had normal hearing on pure-tone audiometry (1000–4000 Hz) and no history of neurological disease, head injury with sequelae, or alcohol/drug dependence within the last 5 years. Groups were matched on age, sex, parental socioeconomic status, and premorbid IQ (Wechsler Abbreviated Scale of Intelligence vocabulary subtest); detailed demographic and clinical characteristics are reported in Lopez-Caballero et al. (2024)^26^. All procedures were approved by the University of Pittsburgh Institutional Review Board (IRB), and all participants provided written informed consent. Research Ethics Board (REB) approval for secondary usage was given by the University of Ottawa Institute of Mental Health Research in Ottawa/Canada.

### Task and stimuli

An auditory oddball paradigm was used. Each recording consisted of 1500 tones presented binaurally at 80 dB with a short stimulus-onset asynchrony (SOA) of 330 ms. Standard tones were 1 kHz, 50 ms (5 ms rise/fall) and occurred in 80% of all trials (1200 tones). To test for the subjects’ processing of tone pitch, pitch deviants were included at 1.2 kHz, 50 ms. Subjects’ processing of tone duration was tested by including duration deviants which were 1 kHz with double length/duration, that is, 100 ms rather than 50 ms (10%; 150 tones). Both deviant tones, e.g., pitch and duration, had the same rise and fall time as the standard tones (5 ms rise/fall). Deviant tones never occurred consecutively and were always separated by at least two standard tones.

Stimuli were delivered via earphones while participants sat in the MEG and watched a silent movie with subtitles, instructed to ignore the sounds of the oddball tones; no behavioral reaction was required as to avoid response-related artifacts (passive listening MMN paradigm).

### MEG acquisition and preprocessing

Data were acquired at the University of Pittsburgh using a 306-channel Elekta Neuromag whole-head system (102 magnetometers and 204 planar gradiometers) in a magnetically shielded room. For some participants, the recording sampling rate was at 1000 Hz, and for the others at 3000 Hz with an online band-pass of 0.1 to 330 Hz. MEG data were preprocessed by in-house scripts using Python (3.11.9) MNE^28^. Electromagnetic noise from outside the helmet was removed using temporal extension of the Signal Space Separation (tSSS)^29,30^. All recordings were resampled to 1000 Hz, and line noise at 60 Hz and harmonics were removed by notch filtering. Data were high-pass filtered at 1 Hz, and finally Independent Component Analysis (ICA) was performed to visually exclude artifacts (blinking, saccades, heartbeat, muscle movement, and noise). All subsequent analysis was performed solely on Magnetometer channels.

### Intertrial phase coherence (ITPC)

Data were epoched to ±1.5 s from the stimulus onset. Artifactual epochs were removed by Autoreject^31^ with default parameters. Cleaned epochs were concatenated and sorted based on their tone (standard, pitch deviant, duration deviant). To account for the minimal trial count differences, for each subject, all three conditions were randomly subsampled to the minimum trial count between the tones (i.e., if a subject had the following trial counts: 1200 standard, 150 pDev, 148 dDev, then all tones are randomly subsampled to 148 trials). The trial count report is as follows: HC: 147.8 ± 3.3, FESZ: 147.2 ± 3.7, FEAFF: 147.5 ± 2.4.

Intertrial phase coherence (ITPC) calculation was performed in a standard manner^9^, time-frequency decomposition was performed by Morlet wavelets using MNE’s internal function (compute_tfr(method = ”morlet”))^28^, with 50 logarithmically spaced frequencies from 3 to 40 Hz. For a balanced temporal and spectral resolution, the number of cycles was set to half of each frequency. For each Subject, ITPC was calculated by averaging complex phase-normalized coefficients across trials per condition.

### Dynamic intertrial phase coherence (dITPC)

First introduced by Lechner et al.^6^, dynamic intertrial phase coherence is similar to normal or static ITPC, with the difference that it is calculated over sliding windows and thus provides information regarding the dynamics of ITPC over time. We computed dITPC on a sliding trial-anchored window corresponding to 55 trials or 18.15 s and an overlap of 50%. The length of the window was chosen so that enough trials remain in the window for a reliable estimation of phase clustering within that window, and also enough windows are available to estimate dynamics over the recording. Only channels in the temporal lobe were selected for this analysis, located by MNE’s internal function (mne.read_vectorview_selection)^28^. For each window, ITPC was calculated using the same criteria at the previous analysis; ITPC values were averaged across 0–330 ms peri-stimulus interval and 3–12.5 Hz frequency band to capture differences between the two groups. The average ITPC values were then assigned to their corresponding windows. The ITPC coefficient of variation (ITPC_CV) for each participant was calculated. To test whether the ITPC_CV results are valid or dependent on the ITPC mean, we performed a confound check by Freedman-Lane permutation. We regressed the ITPC_CV values on the ITPC mean for each subject (and vice versa), and permute the residues 10,000 times to see if the results remain significant independent of each other.

### Mismatch negativity (MMN)

Amplitude MMN was computed from the same equalized epochs used for ITPC (trial counts as reported above), as the deviant-minus-standard difference wave in the auditory region of interest (ROI, the temporal lobe channels), evaluated over the 100–250 ms post-stimulus window, separately for pitch- and duration-deviant conditions. Because standard signed averaging across a bilateral sensor ROI is subject to field cancellation between magnetometers of opposing polarity across hemispheres, channels were first split into left and right hemisphere groups by sensor position. Within each hemisphere, the absolute value of deviant-minus-standard difference was calculated per channel prior to averaging across channels and across the analysis window, yielding a non-negative mean deviance-response magnitude for each hemisphere; the primary amplitude measure was the average of the left- and right-hemisphere magnitudes.

### Intrinsic Neural timescales (INT)–calculation by the knee frequency of the power spectrum (Tau (*τ*))

To avoid the interference of strong oscillations caused by the task possibly affecting the INTs, Intrinsic neural timescales were estimated from the aperiodic “knee” of the power spectrum^16,32,33^ instead of the conventional autocorrelation window (ACW) measured by the autocorrelation function. INTs were estimated using a sliding-window Fitting Oscillatory & One Over F (FOOOF)^27^ analysis on continuous, artifact-cleaned MEG data. Since the slow activity below 1 Hz was required for reliable estimation of the 1/f knee, we used the ICA solutions that were calculated on 1 Hz high-pass filtered data for cleaning the 0.1 Hz high-pass filtered data.

INTs were calculated in a dynamic manner as we segmented the data into 19.8 s windows (19,800 samples) corresponding to 60 tones with 50% overlap. The length of the window is arbitrary and only intended to be long enough for a reasonable PSD calculation and coincide with trial intervals. Windows shorter than 80% of the target length (19,800 data points) were discarded for consistency. Within each retained window, power spectral density (PSD) was computed for all channels using MNE’s internal Welch’s method function (mne.time_frequency.psd_array_welch^32,34^) over 0.1–60 Hz with n_fft = 2048 and 1024-sample overlap. This yielded a PSD for each channel per window.

Each channel-level PSD was parameterized with the FOOOF 1.1.1 toolbox^27^ using the knee aperiodic model (0.1–60 Hz, peak_width_limits = [1,8] Hz, max_n_peaks = 8, min_peak_height = 0.1, peak_threshold = 2.0, aperiodic_mode = ‘knee’). From the aperiodic parameters (offset, knee, exponent), the knee frequency was computed as:

1 $${f}_{k}={{\rm{knee}}}^{(1/{\rm{exponent}})}$$

for knee > 0, and the intrinsic neural timescale as:

2,3,4 $$\tau =1/(2\pi {f}_{k})$$

Pre- and post-knee aperiodic slopes and exponents were derived by masking out peak regions and fitting linear models in log–log space. Quality control was applied per channel per window: FOOOF fits with ${r}^{2}({\rm{coefficient\; of\; determination}})\, < 0.90$, non-finite or outside of the 0.1–25 Hz knee frequencies, and non-finite τ values were rejected. For each window, the median of τ values passing the quality control was picked instead of the mean value to protect against extremes. The same median aggregation was applied to ${f}_{k}$, ${r}^{2}$, and pre/post-knee slopes. In subsequent analysis, the τ values were aggregated by median of windows for each subject for subject level analysis, and were averaged across subjects per dynamic window to create INT time courses. Moreover, calculating the INT for each dynamic window, the INT coefficient of variation (INT_CV) was then calculated for each subject. We also performed the same confound check for INT median and INT_CV as we did for the ITPC results.

### Power spectral density (PSD) and its parametrization

PSD was calculated by the Welch method using MNE’s internal function (mne.time_frequency.psd_array_welch^28^) from 1 Hz to 60 Hz with 2048 as the number of FFT components (frequency resolution ≈ 0.49 Hz at 1000 Hz sampling) and 1024 overlap (50%).

Spectral parameterization was performed on the PSDs using the FOOOF 1.1.1 toolbox^27^. Models were fit to the 1–60 Hz range with the knee aperiodic model (aperiodic_mode = ‘knee’) and the following settings: peak_width_limits = [1.0, 12.0] Hz, max_n_peaks = 8, min_peak_height = 0.0, peak_threshold = 2.0, and other parameters at default values. For each group, FOOOF returned the aperiodic parameters (offset, knee, exponent) and Gaussian peaks. The peak width limit and minimum peak height limits were slightly more relaxed compared to the PSD parameterization for the INT estimation to better capture the periodic activity.

Inter-subject and intra-subject dispersion of PSD spectral profiles were quantified using centroid-based multivariate dispersion measures adapted from permutational analysis of multivariate dispersions (PERMDISP)^35^ applied to FOOOF-derived spectra. For each participant, log10-transformed spectra were represented on a common 1–60 Hz frequency grid separately for the full model, aperiodic component, and periodic component. For inter-subject dispersion, one spectrum per participant, either the average over all channels or the average over the channels on the temporal lobe, was entered into a subject × frequency matrix within each diagnostic group; pairwise Euclidean distances were computed among participants, and principal coordinates analysis (PCoA)^36^ was used to reconstruct Euclidean coordinates from the distance matrix. Group heterogeneity was then quantified as the distance of each participant to the centroid of their respective group in spectral space, yielding one centroid-distance value per subject. For intra-subject dispersion, data were segmented into non-overlapping 16.5 s windows (corresponding to 50 trials), Welch PSDs were computed for each window and averaged across selected channels, and FOOOF was fit to each window to obtain full, aperiodic, and periodic spectra. Euclidean distances were then computed among window-wise spectra within each participant, projected into Euclidean space using PCoA, and summarized as the median distance of windows to the subject-specific centroid, yielding one temporal dispersion value per subject. Group comparisons were performed on these subject-level centroid-distance measures, on the full-spectrum model and aperiodic and periodic components.

### Statistical analysis

The sample size was fixed by the availability of participants in the existing dataset. We therefore performed a sensitivity power analysis to determine the minimum effect size detectable with the available sample. For an independent two-group comparison with 25 FESZ participants and 24 healthy controls, a two-sided α of 0.05 and 80% power, the available sample was sufficient to detect an effect size of approximately Cohen’s d=0.82. Thus, the study was adequately powered to detect large group differences but had limited power for small-to-moderate effects, particularly in correlation and multivariable analyses. All statistical analyses were performed in Python 3.11.9 (NumPy, SciPy, MNE, statsmodels)^28^. Group differences in ITPC were tested using MNE’s spatio-temporal cluster permutation test^28^. In all the group comparisons, since FESZ and HC groups were age- and sex matched, they were not accounted for as covariates. Subject ITPC maps were cropped to a common time–frequency window (−1 to 1 s, 3–40 Hz). A channel adjacency matrix was derived from the MEG sensor layout and combined with frequency and time dimensions to form a 3D adjacency (time × frequency × channel). Since we had a clear hypothesis of decreased ITPC in FESZ, a fixed cluster-forming threshold of *t* = 2.41 (one-sided *p* ≈ 0.01) and 3000 permutations were used, with cluster mass as the test statistic. Since we compared three conditions (tones) from the same subjects, we applied Bonferroni correction, so *p* < 0.0167 for clusters was considered significant. Group differences in MMN amplitude were assessed using the Mann–Whitney U test with Bonferroni correction across the two deviant conditions.

For intrinsic timescales (*τ*) and intrasubject *τ* variability, group differences were assessed with a regression-based Freedman–Lane permutation test (10,000 permutations) controlling for goodness of the FOOOF model fit for tau values, and goodness of fit and window count for intrasubject variability of tau: Each metric was log(1 + x)-transformed, z-scored, and entered as the outcome variable in a linear model including the specified covariates and diagnostic group as predictors. Two-sided *p*-values were obtained from the permutation distribution, with *α* = 0.05.

For both inter-subject and intra-subject dispersion analyses, statistical tests were performed using subject-level permutation testing. The full-spectrum model was specified as the primary outcome. Only when the full-spectrum comparison was significant were follow-up permutation tests performed on the aperiodic and periodic components. To account for multiple testing at the follow-up stage, Bonferroni correction was applied across the two decomposition tests.

### Correlation analyses

Relationships among the primary neural measures (ITPC, ITPC temporal coefficient of variation, INT median, INT temporal coefficient of variation, intra-subject PSD dispersion, and duration-deviant MMN amplitude) were examined by Spearman correlations separately in HC and FESZ groups. Multiple comparisons were corrected with Benjamini–Hochberg false discovery rate (FDR) within each group across the 10 pairs (*q* < 0.05).

Duration-deviant MMN amplitude, reflecting the evoked amplitude response rather than an ongoing dynamic, was analyzed separately in relation to each of the five background measures, with FDR correction applied within each group across this second, five-test family (*q* < 0.05).

Relationships between the six primary neural measures and clinical symptoms measured using the Positive and Negative Syndrome Scale (PANSS) and antipsychotic dosage (chlorpromazine-equivalent) were tested in FESZ only using Spearman correlation. FDR correction was applied within each family (12 correlation tests for symptoms (6 measures × 2 symptom scores), 6 tests for medication, *q* < 0.05)).

## Results

### Decreased mismatch negativity in FESZ

**Fig. 2:**
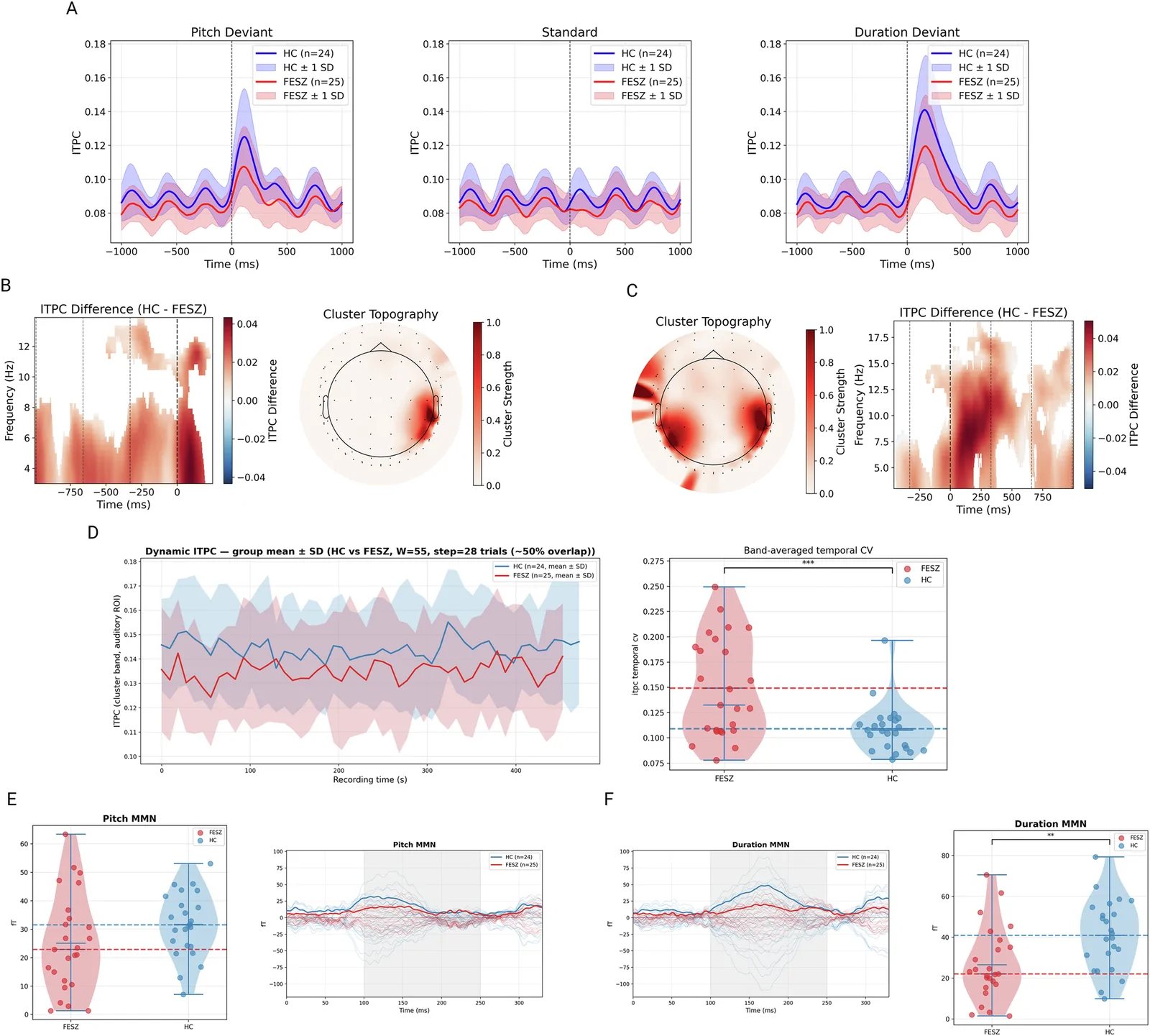
Global intertrial phase coherence (ITPC) difference between healthy (HC) and first-episode schizophrenia (FESZ) subjects with significant clusters & MMN results. **A** Global ITPC time course ± 1 s from the stimulus onset averaged across all channels and all frequencies. Blue shows Healthy intertrial phase coherence (ITPC) ± SD, and red indicates First-Episode Schizophrenia ITPC ± SD. **B** Time-frequency trajectory of ITPC difference between groups in the pitch deviant cluster and topomap of cluster strength. Solid dashed line corresponds to the tone onset, and faded dashed lines mark pre- and post-tones. **C** Time-frequency trajectory of ITPC difference between groups in the duration deviant cluster and topomap of cluster strength. Solid dashed line corresponds to the tone onset, and faded dashed lines mark pre- and post-tones. **D** Dynamic ITPC over sliding windows of 18.15 s (55 trials) and 50% overlap. ITPC value for each window is the average across the 3–12.5 Hz frequency band. Group differences in the ITPC_CV are shown in the violin plot. **E** Pitch MMN grand-average waveforms and group amplitude distributions (HC *n* = 24, FESZ *n* = 25); no significant group difference**. F** Duration MMN waveforms and amplitude distributions; FESZ showed significantly reduced duration MMN vs HC (**, *p* < 0.01, *d* = 0.82).

To replicate previous findings, we first calculated the evoked potential typical for the oddball paradigm, the mismatch negativity (Fig. 2E, F). Averaged mismatch negativity (MMN) across left and right hemispheres was decreased in FESZ for both pitch (HC mean: +31.7 fT, FESZ mean: +25.0 fT, HC − FESZ: +6.6 fT, *d* = +0.46, p_MW = 0.0767) and duration (HC mean: +40.8 fT, FESZ mean: +26.5 fT, HC − FESZ: +14.3 fT, *d* = +0.82, p_MW = 0.0056) deviant tones, but the reduction was only significant in the duration deviant tones before and after Bonferroni correction. This is consistent with the original results on this dataset^26^. Because relatively few deviant trials were available within short sliding windows, dynamic MMN estimates could not be calculated with sufficient reliability, that is, its variability as in SD or CV.

### Decreased phase coherence (ITPC) and increased phase coherence temporal variability (ITPC_CV)–temporal imprecision in the phase domain

Intertrial Phase Coherence (ITPC) was visibly reduced in FESZ compared to HC group in all three conditions, standard, pitch, and duration deviant (Fig. 2A). However, only the deviant tones resulted in significant decrease of ITPC in FESZ compared to HC. The significant pitch deviant cluster (Fig. 2B) spanned from −999 to +249 ms from the tone onset, 3–13.9 Hz in frequency, and 46 channels, with the strongest located on the right auditory cortex (cluster *p* = 0.0087, Cohen’s *d* = 1.755; HC mean ITPC = 0.1518, FESZ mean ITPC = 0.0947). The duration deviant significant cluster (Fig. 2C) (cluster *p* = 0.0007, Cohen’s *d* = 2.098; HC mean ITPC = 0.1621, FESZ mean ITPC = 0.1030) was broader in time (−459 to +999 ms) and frequency (3–19.1 Hz) and spanned across more (100) channels mainly on the left and right auditory cortex.

Dynamic ITPC showed greater coefficient of variation in FESZ compared to the healthy group (Fig. 2D) (HC ITPC_CV = 0.109, FESZ ITPC_CV = 0.149, Cohen’s *d* (HC - FESZ) = −1.03, *p* (perm) = 0.001). ITPC_CV difference between the groups remains significant after accounting for ITPC mean as a covariate (FL *p* = 0.011). This suggests that increased temporal variability in FESZ is not just a byproduct of poor ITPC mean with lower values.

Together, these findings replicate previous findings of both reduced ITPC in FESZ^1,5,6,10^ and increased ITPC temporal variability^6^. While extending them by showing ITPC reduction and higher ITPC temporal variability to also hold (i) in first episode schizophrenia, and (ii) ITPC reduction in specifically the duration rather than pitch-deviant tones, as they elicited the strongest ITPC reduction in FESZ. This indicates increased temporal imprecision in FESZ with respect to its own phase dynamics (phase coherence) at both specific time points (e.g., reduced mean ITPC) and between time points (e.g., increased ITPC-CV) during specifically the processing of temporally deviant inputs (e.g., the duration deviant as distinguished from pitch deviant).

### Prolonged and more variable timescales (INT)–temporal imprecision in the time domain

**Fig. 3:**
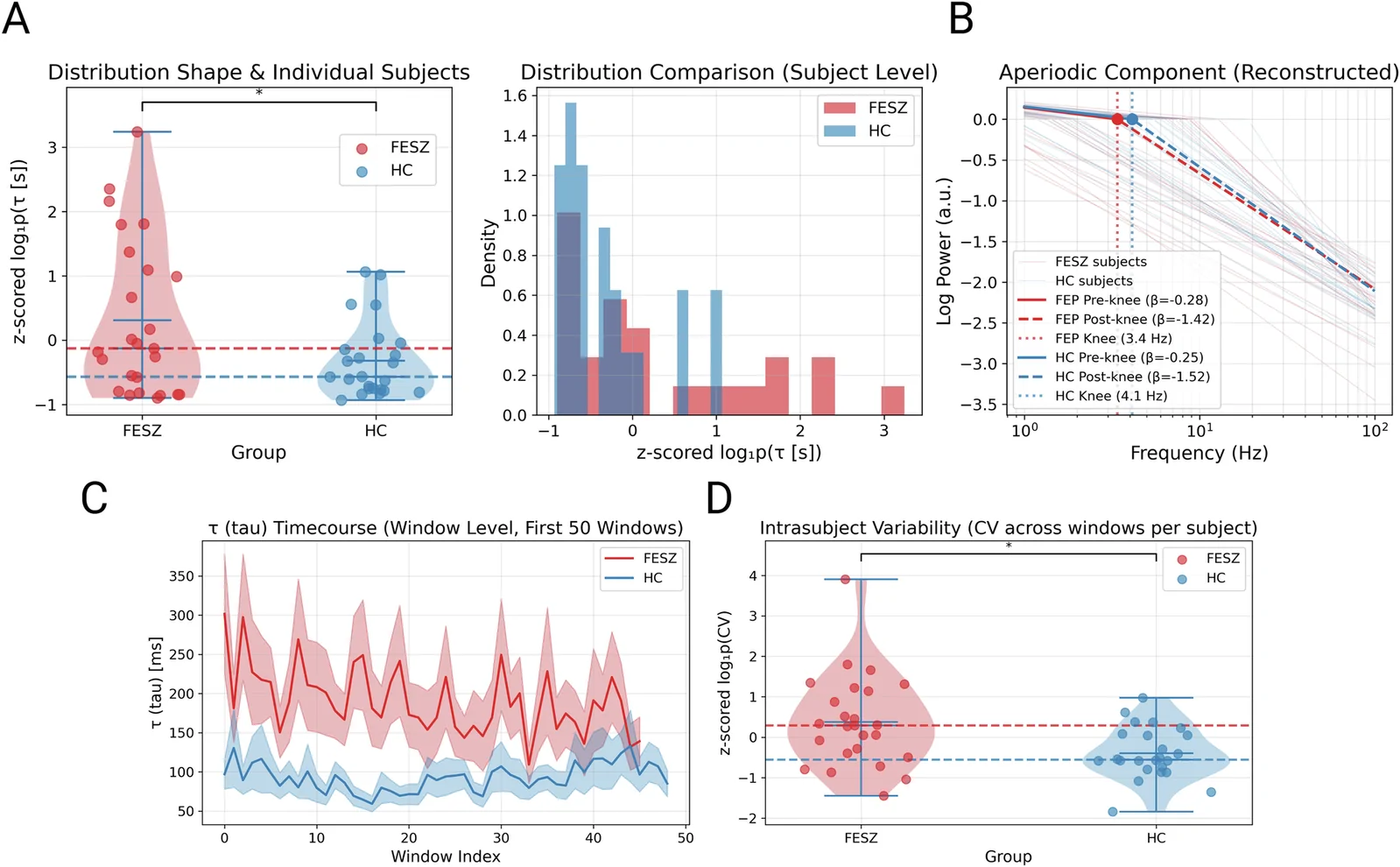
Intrinsic neural timescale (INT) estimation. **A** Distribution shape and comparison for the power spectrum’s knee-based calculation of the timescale, e.g., *τ* ms for HC and FESZ subjects. Each dot represents the z-scored median *τ* for one subject, and the dashed lines mark the group averages. **B** Group median FOOOF aperiodic fit log-log pre- and post-knee slopes and knee frequency. **C** Dynamic *τ* ms time course over 50 19.8 s windows. *τ* estimates were derived from 46.0 windows per FESZ subject (range 14–50) and 49.7 windows per control subject (range 49–50). **D** *τ* coefficient of variation measured by each subject’s standard deviation (SD) divided by mean over 50 windows. (* : *p* < 0.05).

We next investigated the INTs, which were measured through the knee of the power spectrum, e.g., the tau (*τ*). Dynamic INT median showed significant increase with longer INT in the FESZ group (166.0 ± 163.9 ms, *n* = 25) compared to healthy subjects (80.8 ± 72.0 ms, *n* = 24)(Fig. 3A). The difference remained significant after adjusting for FOOOF model goodness of fit using a Freedman–Lane permutation test for tau (two-sided *p* = 0.0316, partial Cohen’s *d* = 0.652). This suggests prolonged INT median in FESZ; such prolonged duration in the length of the temporal windows of INT suggests temporal imprecision during input processing as required in our task.

The temporal variability of *τ* (CV across INT windows) was also significantly (two-sided *p* = 0.0123, partial Cohen’s *d* = 0.752) higher after adjusting for goodness of fit and window count in FESZ (mean CV 0.8133 ± 0.3445) than in controls (0.6245 ± 0.2854) (Fig. 3D). On average, τ estimates were derived from 46.0 windows per FESZ subject (range 14–50) and 49.7 windows per control subject (range 49–50). However, unlike ITPC_CV, INT_CV did not survive after controlling for INT level, as it reduced to a trend (FL *p* = 0.0669).

Together, these findings replicate previous results of prolonged INT mean in schizophrenia^10,20^ and extend them by (i) showing a similar INT median prolongation in first-episode patients, e.g., FESZ; (ii) the calculation of dynamic INT showing higher variability in FESZ; and (iii) high inter-subject differentiation of HC and FESZ. Together, these findings indicate increased temporal instability and imprecision in the durations themselves (longer INT median) and their variance over time (INT-CV) in FESZ.

### More dispersed power spectral density (PSD) with higher within-group and within-subject variability – Temporal imprecision in the frequency domain

**Fig. 4:**
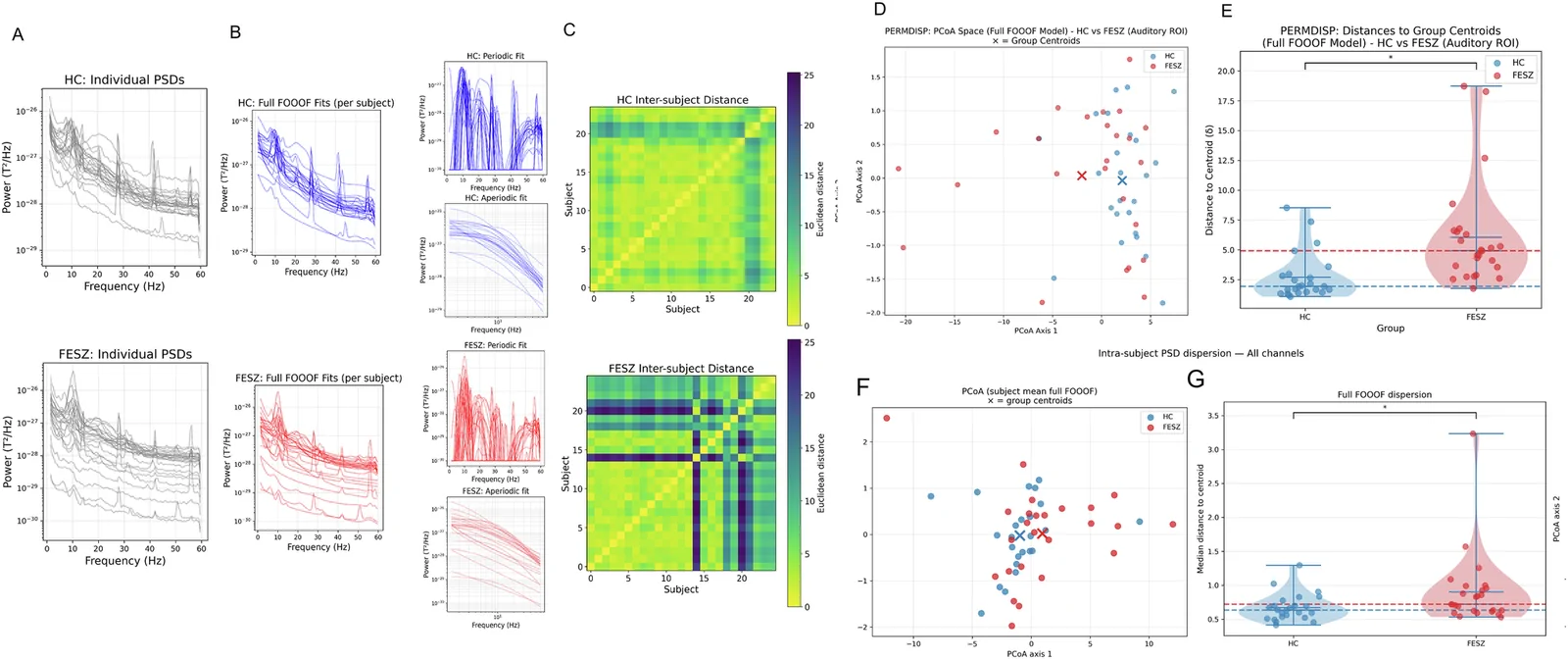
Inter-subject and intra-subject PSD dispersion of FOOOF-derived spectral profiles in HC and FESZ. **A**–**D** Show the methodological steps that are shared between the intra- and inter-subject dispersion analysis. The difference, is that for the inter-subject analysis each PSD belongs to one subject, whereas in the intra-subject analysis each PSD belongs to one window of one subject, and at the end, in the inter-subject analysis the distance to centroid is plotted as one data point representing one subject’s distance to group centroid, whereas in the intra-subject analysis the median of window distances will be recorded for each participant. **A** Individual power spectral densities (PSDs) for healthy controls (HC) and first-episode schizophrenia (FESZ). Subject-level FOOOF-derived spectra, showing full-model fits and their decomposition into periodic and aperiodic components for each group. **C** Inter-subject Euclidean distance matrices computed from full-spectrum FOOOF profiles. **D** Principal coordinates analysis (PCoA) of the inter-subject distance matrix in the auditory ROI, with each point representing one participant and crosses indicating group centroids. **E** Distance-to-centroid values from the inter-subject analysis in the auditory ROI for the full FOOOF model. **F** Principal coordinates analysis (PCoA) of the intra-subject distance matrix with all channels, with each point representing the median of window distances and crosses indicating subject centroids. **G** Intra-subject temporal PSD dispersion across non-overlapping windows for all channels, quantified as the median distance of window-wise FOOOF spectra to the subject-specific centroid for the full power spectrum (* : *p* < 0.05).

To investigate input processing in the frequency domain, we examined within-subject and within-group heterogeneity of subjects’ PSDs. The intra-subject dispersion of FESZ participants was significantly higher than that of the healthy group (diff (HC-FESZ) = −0.2321, *p* = 0.0242, *d* = −0.564) (Fig. 4F, G). Follow-up tests on the components of the power spectrum showed that the higher dispersion in the FESZ participants is mainly driven by the aperiodic component of the power spectra (p_Bonf = 0.0476, *d* = −0.532), rather than the periodic component (p_Bonf = 1.00, *d* = 0.191) (Supplementary Fig. 2).

Since the input was the same for all the participants, we also sought to investigate inter-subject group variability effects in the Power spectra. At the scope of all channels, there were no significant differences between the HC and FESZ groups, but when restricted to the channels on the temporal lobes where the auditory input is processed, the FESZ group showed significantly higher dispersion compared to the HC group (*p* = 0.031497) (Fig. 4D, E). The group difference of the aperiodic (*p* = 0.07) and the periodic (*p* = 0.19) components was not significant; however, the difference in the aperiodic component was higher.

Together, these results indicate that PSD profiles were more heterogeneous and showed greater temporal dispersion in FESZ, indicating less stable spectral organization during the auditory task, especially for the aperiodic 1/*f* background.

### Relating phase-, time-, and frequency domains–convergent temporal imprecision in schizophrenia

**Fig. 5:**
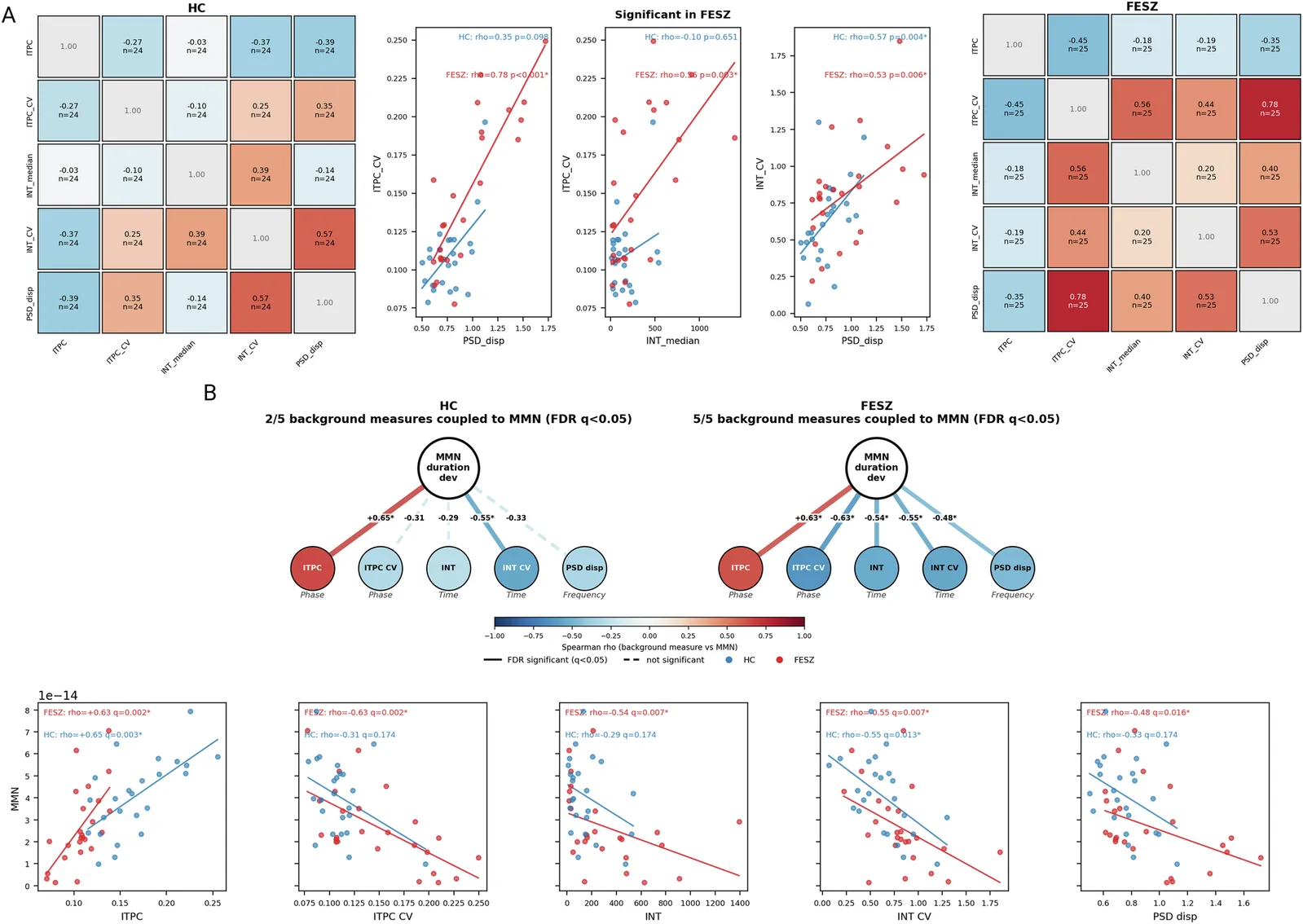
Correlations among primary dynamic neural measures and Background domain instability in relation to MMN amplitude. **A** HC vs FESZ. Left and right matrices show pairwise Spearman correlations among the five primary background measures—ITPC, ITPC temporal coefficient of variation (CV), INT (median), INT temporal CV, and intra-subject PSD dispersion—computed separately in HC (left, *n* = 24) and FESZ (right, *n* = 25). Off-diagonal cells show Spearman ρ and sample size; diagonal cells are uninformative (*ρ* = 1) and shown as plain reference cells. Cell shading follows a red–blue gradient scaled to *ρ* (red = positive, blue = negative). Correlations surviving Benjamini–Hochberg FDR correction (*q* < 0.05) are highlighted. Center panels show scatter plots with per-group linear trend lines for pairs reaching FDR significance in FESZ (top row); the only significant pair in HC can also be seen in the INT_CV – PSD disp plot; each panel reports Spearman *ρ* and p-value for both groups, with an asterisk marking the group(s) in which that pair survived FDR correction. **B** *Top:* The association between each of the five primary background measures (ITPC, ITPC temporal coefficient of variation (CV), INT [median], INT temporal CV, PSD dispersion) and duration-deviant MMN amplitude, in HC (left, *n* = 24) and FESZ (right, *n* = 25). Each background measure is connected to MMN by an edge whose color reflects the Spearman *ρ* (red–blue gradient, scale shown below) and whose line style indicates whether that association survived Benjamini–Hochberg FDR correction within the five-test family (solid = *q* < 0.05; dashed = not significant); edge thickness scales with |*ρ*|. Node fill color follows the same ρ gradient as its edge. The italicized label beneath each node indicates its signal domain (phase, time, or frequency). Two of five background measures were significantly associated with MMN in HC; all five were significant in FESZ. *Bottom:* Corresponding scatter plots for each background measure against MMN amplitude, with both groups overlaid (HC, blue; FESZ, red) and per-group linear trend lines. Spearman ρ and FDR-corrected q-value are reported for each group; asterisk denotes *q* < 0.05.

Are the changes in all three signal domains in SZ related to each other? To address this question, we conducted correlation analyses between the measures of the three signal domains (Fig. 5A). Correlations among the five dynamic measures (ITPC, ITPC-CV, INT median, INT temporal CV, intra-subject PSD dispersion) were sparse in HC, with only 1 of 10 pairs surviving FDR correction (INT-CV – PSD dispersion, *ρ* = 0.57, *q* = 0.035). In FESZ, 3 of 10 pairs survived correction, spanning all three domains: ITPC- CV – PSD dispersion (*ρ* = 0.78, *q* < 0.001), ITPC-CV – INT median (*ρ* = 0.56, *q* = 0.018), and INT-CV – PSD dispersion (*ρ* = 0.53, *q* = 0.020). The INT-CV – PSD dispersion association was present in both groups; the two associations involving ITPC-CV were specific to FESZ. All significant dynamic associations were positive, indicating that greater instability in one domain was accompanied by greater instability in others, a relationship largely absent in HC.

### Relationship of the three signal domains’ measures with the MMN amplitude

Finally, we also related the measures of the three signal domains to the MMN, a well established standard measure of SZ. In HC, MMN duration-dev amplitude was significantly associated with only 2 of 5 dynamic measures after FDR correction (Fig. 5B) : ITPC level (*ρ* = 0.65, *q* = 0.003) and INT temporal CV (*ρ* = −0.55, *q* = 0.013). In FESZ, all 5 dynamic measures were significantly associated with MMN duration-dev amplitude (all *q* ≤ 0.016): ITPC level (*ρ* = 0.63, *q* = 0.002), ITPC-CV (*ρ* = −0.63, *q* = 0.002), INT median (*ρ* = −0.54, *q* = 0.007), INT-CV (*ρ* = −0.55, q = 0.007), and PSD dispersion (*ρ* = −0.48, *q* = 0.016). Greater intertrial phase coherence (ITPC) in the duration deviant tones was associated with larger MMN duration-dev amplitude in both groups. In contrast, greater instability across every dynamic domain (phase, time, and frequency) was associated with a smaller MMN response, an association present for only one instability measure in HC (INT- CV) but for all three (ITPC-CV, INT-CV, intra-subject PSD dispersion) in FESZ.

### Clinical and medication associations

No neural measure survived FDR correction for association with PANSS positive or negative symptom scores, or with antipsychotic dosage, in FESZ (symptoms: all *q* ≥ 0.28; medication: all *q* ≥ 0.66). The lowest nominal p-value in either family was for INT (median) with PANSS positive symptom score (*ρ* = 0.45, *p* = 0.023, *q* = 0.28), which did not approach significance after correction, though (Supplementary Fig. 1).

## Discussion

### Temporal imprecision in phase, time, and frequency domains

We demonstrate temporal imprecision during input processing in all three domains of the MEG signal: phase, time, and frequency.

Our first finding, decreased ITPC in first-episode schizophrenia, supports previous evidence^1,5,6,10^, and extends it by showing decreased ITPC in early stages of the disorder specifically in the duration deviant oddball task. This finding further supports the temporal imprecision hypothesis of input processing in the phase domain by showing the inability of the brains of individuals with schizophrenia to precisely tune its phase angle to the temporally variable input (e.g., the duration deviant tone). This imprecision highlights impaired entrainment: if the brain is not able to tune itself to the exact timing of the external stimuli, then the temporal segregation of the subsequently incoming external stimuli from each other as well as its differentiation from the ongoing internal activity are both compromised. This is further supported by the increase in the temporal variance of intertrial phase coherence over the length of the recording as it suggests the impairment is sustained through time, that is, between different time points. Our confirmatory MMN results also support this pattern as only MMN of the duration deviant tones were reliably reduced in the FESZ participants as opposed to less attenuated MMN of the pitch deviant tones.

Our second finding, prolonged and more variable INT, also highlights temporal imprecision of input processing in the time domain. INTs exhibit a hierarchy from short to long that is believed to be key in temporally integrating and segregating different inputs^11,12,17^. Longer INT means that different inputs, which are usually temporally segregated, may be lumped and thus processed together, e.g., temporal integration—this indicates temporal imprecision in the time domain. Our results support earlier evidence of prolongation of INTs in schizophrenia as measured by autocorrelation window (ACW) in rest and task in EEG^10,20^. Moreover, our results show high intrasubject variability of INTs in the schizophrenia patients over the duration of the recording. This high variability further supports the temporal imprecision hypothesis: if the INTs are extremely variable in their duration or length, the processing of inputs will be affected by the strong changes in their length, such that even rhythmic inputs, like in our oddball tasks, cannot be followed and neurally encoded in a correspondingly rhythmic way.

Our third main finding was increased intra- and inter-subject dispersion within the power spectral density (PSD) in FESZ, indicating that spectral profiles are less stereotyped and rhythmic across individuals with schizophrenia than in healthy controls. This effect was strongest for within-subject dispersion and for the aperiodic 1/f component across all channels, which shows increased temporal variability in the frequency domain of the brain signal. However, it only became significantly detectable for the inter-subject dispersion when restricting analyses to channels in the temporal ROI, reflecting those channels primarily involved in the processing of the auditory input in our oddball paradigm. Because all participants received identical auditory tones and the same silent movie, elevated dispersion in the PSD suggests greater variability and thus spectral or temporal imprecision in FESZ, indicating less stable spectral organization during the auditory task.

### Temporal imprecision holds in all three signal domains–basic disturbance of schizophrenia

The observation of temporal imprecision in all three signal domains all at once suggests that none are occurring in isolation; indeed, our results show a positive correlation between the temporal variability in all three, phase, time, and frequency domains. This was stronger in our FESZ subjects: there were more significant correlations among the different measures in FESZ than in HC. Moreover, the strong negative correlation of all the variability measures across the dynamic domains to MMN, of which only one holds in the HC groups, and its strong negative correlation to the length of the INTs, suggests the impairment of MMN response in the first episode schizophrenia patients may be due to the convergent temporal imprecision in all the dynamic domains of the signal, a feature that is largely absent in the HC group.

Together, these findings show a close relationship among the different measures, especially FESZ; this suggests that temporal imprecision may occur and reverberate across the different signal domains and their respective dynamics. Further, we observed that the temporal imprecision in these measures strongly couples with the MMN by reducing its amplitude, a hallmark finding in FESZ. We therefore suggest that the temporal imprecision in our measures of the three signal domains, frequency, time, and phase, may provide the neurodynamic background for the MMN and its amplitude reduction in the foreground. That supports the assumption that temporal imprecision may provide the basic disturbance of schizophrenia, holding across all three signal domains and shaping its most visible surface or foreground manifestation, the MMN.

Our temporal imprecision model aligns well with prior schizophrenia literature reporting altered temporal organization of intrinsic activity that is indexed by reduced long-range temporal correlations^24^ and temporal dysconnectivity^23^ and robust reductions in phase-consistent synchronization to rhythmic auditory structure^37^. This implies reduced reliability of temporal coordination in schizophrenia that is manifest as more heterogeneous spectral signatures at the individual and group level. Finally, temporal imprecision is in line with other hypotheses of schizophrenia such as imprecision in predictive coding^38^.

### Future directions

Future studies could explore the differentiation of schizophrenia and other affective disorders using the INTs and the characterization of the PSD with larger sample sizes. Computational modeling has been used before in the framework of temporal irregularity driving predictive failure in schizophrenia^39^, and other neural mass models explaining abnormal beta-rebound in schizophrenia^40^, but now models could be used to test the causal interactions among temporal imprecision across the three signal domains in a mechanistic manner. Also, given that temporal fragmentation is a defining perceptual feature of time experience in schizophrenia, and recent development of a rating scale for time experience in schizophrenia (STEP scale^41^), future studies can explore whether temporal imprecision of the input processing in schizophrenia is related to the experience of temporal fragmentation in both thought and perception. This is in accordance with the assumption that time and space deficits have been suggested to serve as proto-phenomenal background, giving rise perceptual and behavioral changes and symptoms^42^; investigating how the dynamic measures relate to time and space experience could further shine light on their relationship to symptoms. Another line of investigation could explore temporal imprecision in different domains of the brain signal during the resting state compared to the task state.

### Limitations

The present study has several limitations. First, sample sizes are modest (25 FESZ, 24 HC), which makes the multimeasure analysis underpowered, and longitudinal diagnostic follow-up was not available in the secondary dataset; therefore, the stability of the initial schizophrenia-spectrum diagnoses could not be evaluated. Second, PSD parameterization and knee-based INT estimation depend on preprocessing and model choices (e.g., frequency range, window length, filtering, peak settings, quality-control thresholds), and while we applied strict quality-control criteria, residual sensitivity to signal-to-noise differences and fit stability may contribute to variability and group effects. Third, while the cohort was restricted to early-stage patients with limited antipsychotic exposure, and the provided information on symptoms and medication dosage did not show any reliable correlations to the results, clinical heterogeneity (symptom profiles, medication status, comorbidity, sleep/stress) remains and may contribute to increased inter-subject variability. Further, analyses were performed on magnetometer channels only; although this increases methodological consistency, source-level analyses would be required to strengthen anatomical interpretation and to test whether the observed effects localize to specific cortical generators. Finally, we and others have shown prolonged INT in EEG in FESZ. This contrasts with the results of shortened INTs in schizophrenia in slower frequencies measured by fMRI^43–46^; this discrepancy remains yet to be investigated^43^. One possible explanation could be that the shortening of INTs in slow frequencies in the BOLD signal and the prolongation of the INTs in faster frequencies in the MEG and EEG signal may point to a dedifferentiation and thus impairment of the hierarchy of INTs.

## Conclusion

We have here demonstrated further evidence of convergent temporal imprecision in first-episode schizophrenia in the three major domains of the brain MEG signal: phase, time, and frequency. We extend previous evidence on temporal imprecision in phase domain by showing reduced intertrial phase coherence (ITPC) in auditory oddball task, specifically in response to the duration deviant tone in first-episode patients, in time domain by showing prolonged and more variable INTs as measured by knee frequency of the aperiodic component of the power spectral density (PSD) during task state, and more heterogeneous power spectral density (PSD) as measured by intra- and inter-subject spectral dispersion suggesting higher variability in the PSD of first-episode schizophrenia patients. Finally, we have shown positive correlation between temporal variability in all three signal domains, including their association to impaired mismatch negativity in first-episode schizophrenia. These findings further support the model of temporal imprecision constituting a basic disturbance in schizophrenia, holding across all three signal domains during input processing.

## Supplementary information

Supp Material

## Data Availability

Data is publicly available through an OpenNeuro repository (OpenNeuro Accession Number: ds004837).

## Code availability

Code is available upon reasonable request.

## References

[1] Wolff, A. & Northoff, G. Temporal imprecision of phase coherence in schizophrenia and psychosis—dynamic mechanisms and diagnostic marker. *Mol. Psychiatry* **29**, 425–438 (2024). 10.1038/s41380-023-02337-z 38228893

[2] Javitt, D. C. & Sweet, R. A. Auditory dysfunction in schizophrenia: integrating clinical and basic features. *Nat. Rev. Neurosci.* **16**, 535–550 (2015). 10.1038/nrn4002 PMC4692466 26289573

[3] Javitt, D. C. & Freedman, R. Sensory processing dysfunction in the personal experience and neuronal machinery of schizophrenia. *Am. J. Psychiatry* **172**, 17–31 (2015). 10.1176/appi.ajp.2014.13121691 PMC4501403 25553496

[4] Carroll, C. A., O’Donnell, B. F., Shekhar, A. & Hetrick, W. P. Timing dysfunctions in schizophrenia span from millisecond to several-second durations. *Brain Cogn.* **70**, 181–190 (2009). 10.1016/j.bandc.2009.02.001 19282082

[5] Wolff, A. et al. It’s in the timing: reduced temporal precision in neural activity of schizophrenia. *Cereb. Cortex* **32**, 3441–3456 (2022). 10.1093/cercor/bhab425 PMC9376870 34875019

[6] Lechner, S. et al. Temporal imprecision and its dynamics in schizophrenia. *Translat. Psychiatry* **15**, 279 (2025). 10.1038/s41398-025-03510-4 PMC12350930 40804052

[7] O’Donnell, B. F. et al. The auditory steady-state response (ASSR): a translational biomarker for schizophrenia. *Suppl. Clin. Neurophysiol.* **62**, 101–112 (2013). 10.1016/b978-0-7020-5307-8.00006-5 PMC4959266 24053034

[8] Lakatos, P., Gross, J. & Thut, G. A new unifying account of the roles of neuronal entrainment. *Curr. Biol.* **29**, R890–R905 (2019). 10.1016/j.cub.2019.07.075 PMC6769420 31550478

[9] Cohen, M. X. Intertrial phase clustering. *Anal. Neural Time Ser. Data* 261–278 10.7551/MITPRESS/9609.003.0024 (2014).

[10] Lechner, S. & Northoff, G. Prolonged intrinsic neural timescales dissociate from phase coherence in schizophrenia. *Brain Sci.* **13**, 695 (2023). 10.3390/brainsci13040695 PMC10137081 37190660

[11] Wolff, A. et al. Intrinsic neural timescales: temporal integration and segregation. *Trends Cogn. Sci.* **26**, 159–173 (2022). 10.1016/j.tics.2021.11.007 34991988

[12] Golesorkhi, M. et al. The brain and its time: intrinsic neural timescales are key for input processing. *Commun. Biol*. **4**, 970 (2021). 10.1038/s42003-021-02483-6 PMC8368044 34400800

[13] Hasson, U., Chen, J. & Honey, C. J. Hierarchical process memory: memory as an integral component of information processing. *Trends Cogn. Sci.* **19**, 304–313 (2015). 10.1016/j.tics.2015.04.006 PMC4457571 25980649

[14] Murray, J. D. et al. A hierarchy of intrinsic timescales across primate cortex. *Nat. Neurosci.* **17**, 1661–1663 (2014). 10.1038/nn.3862 PMC4241138 25383900

[15] Honey, C. J. et al. Slow cortical dynamics and the accumulation of information over long timescales. *Neuron* **76**, 423–434 (2012). 10.1016/j.neuron.2012.08.011 PMC3517908 23083743

[16] Çatal, Y. & Northoff, G. IntrinsicTimescales.jl: A Julia package to estimate intrinsic (neural) timescales (INTs) from time-series data. *J. Open Source Softw.* **10**, 8261 (2025).

[17] Golesorkhi, M., Gomez-Pilar, J., Tumati, S., Fraser, M. & Northoff, G. Temporal hierarchy of intrinsic neural timescales converges with spatial core-periphery organization. *Commun. Biol.* **4**, 277 (2021). 10.1038/s42003-021-01785-z PMC7933253 33664456

[18] Himberger, K. D., Chien, H. Y. & Honey, C. J. Principles of temporal processing across the cortical hierarchy. *Neuroscience* **389**, 161–174 (2018). 10.1016/j.neuroscience.2018.04.030 29729293

[19] Chen, J. et al. Shared memories reveal shared structure in neural activity across individuals. *Nat. Neurosci.* **20**, 115–125 (2016). 10.1038/nn.4450 PMC5191958 27918531

[20] Northoff, G., Sandsten, K. E., Nordgaard, J., Kjaer, T. W. & Parnas, J. The self and its prolonged intrinsic neural timescale in schizophrenia. *Schizophr. Bull.* **47**, 170–179 (2021). 10.1093/schbul/sbaa083 PMC7825007 32614395

[21] Redwan, S. M., Uddin, M. P., Ulhaq, A., Sharif, M. I. & Krishnamoorthy, G. Power spectral density-based resting-state EEG classification of first-episode psychosis. *Sci. Rep.* **14**, 15154 (2024). 10.1038/s41598-024-66110-0 PMC11219808 38956297

[22] Hasanaj, G. et al. Periodic and aperiodic alterations of resting-state EEG in schizophrenia spectrum disorders: cognitive and clinical insights. *Eur. J. Neurosci.* **62**, e70263 (2025). 10.1111/ejn.70263 PMC12495554 41045101

[23] Alamian, G. et al. Patient, interrupted: MEG oscillation dynamics reveal temporal dysconnectivity in schizophrenia. *Neuroimage Clin.* **28**, 102485 (2020). 10.1016/j.nicl.2020.102485 PMC7691748 33395976

[24] Nikulin, V. V., Jönsson, E. G. & Brismar, T. Attenuation of long-range temporal correlations in the amplitude dynamics of alpha and beta neuronal oscillations in patients with schizophrenia. *Neuroimage* **61**, 162–169 (2012). 10.1016/j.neuroimage.2012.03.008 22430497

[25] Northoff, G. & Hirjak, D. Spatiotemporal psychopathology – an integrated brain-mind approach and catatonia. *Schizophr. Res.* **263**, 151–159 (2024). 10.1016/j.schres.2022.10.006 36335076

[26] López-Caballero, F., Curtis, M., Coffman, B. A. & Salisbury, D. F. Is source-resolved magnetoencephalographic mismatch negativity a viable biomarker for early psychosis?. *Eur. J. Neurosci.* **59**, 1889–1906 (2024). 10.1111/ejn.16107 PMC10837325 37537883

[27] Donoghue, T. et al. Parameterizing neural power spectra into periodic and aperiodic components. *Nat. Neurosci.* **23**, 1655–1665 (2020). 10.1038/s41593-020-00744-x PMC8106550 33230329

[28] Larson, E. et al. MNE-Python. 10.5281/ZENODO.14519545.

[29] Taulu, S., Simola, J. & Kajola, M. Clinical applications of the signal space separation method. *Int. Congr. Ser.* **1270**, 32–37 (2004).

[30] Taulu, S. & Simola, J. Spatiotemporal signal space separation method for rejecting nearby interference in MEG measurements. *Phys. Med. Biol*. **51**, 1759–68 (2006). 10.1088/0031-9155/51/7/008 16552102

[31] Jas, M., Engemann, D. A., Bekhti, Y., Raimondo, F. & Gramfort, A. Autoreject: automated artifact rejection for MEG and EEG data. *Neuroimage* **159**, 417–429 (2017). 10.1016/j.neuroimage.2017.06.030 PMC7243972 28645840

[32] Chaudhuri, R., He, B. J. & Wang, X. J. Random recurrent networks near criticality capture the broadband power distribution of human ECoG dynamics. *Cereb. Cortex* **28**, 3610–3622 (2018). 10.1093/cercor/bhx233 PMC6132289 29040412

[33] Gao, R., Van den Brink, R. L., Pfeffer, T. & Voytek, B. Neuronal timescales are functionally dynamic and shaped by cortical microarchitecture. *eLife* **9**, 1–44 (2020). 10.7554/eLife.61277 PMC7755395 33226336

[34] Welch, P. D. The use of fast Fourier transform for the estimation of power spectra: a method based on time averaging over short, modified periodograms. *IEEE Trans. Audio Electroacoust.* **15**, 70–73 (1967).

[35] Anderson, M. J. Distance-based tests for homogeneity of multivariate dispersions. *Biometrics* **62**, 245–253 (2006). 10.1111/j.1541-0420.2005.00440.x 16542252

[36] Gower, J. C. Some distance properties of latent root and vector methods used in multivariate analysis. *Biometrika* **53**, 325 (1966).

[37] Thun, H., Recasens, M. & Uhlhaas, P. J. The 40-Hz auditory steady-state response in patients with schizophrenia: a meta-analysis. *JAMA Psychiatry* **73**, 1145–1153 (2016). 10.1001/jamapsychiatry.2016.2619 27732692

[38] Liddle, P. F. & Liddle, E. B. Imprecise predictive coding is at the core of classical schizophrenia. *Front. Hum. Neurosci.* **16**, 818711 (2022). 10.3389/fnhum.2022.818711 PMC8928728 35308615

[39] Karanikolaou, M., Limanowski, J. & Northoff, G. Does temporal irregularity drive prediction failure in schizophrenia? Temporal modelling of ERPs. *Schizophrenia* **8**, 23 (2022). 10.1038/s41537-022-00239-7 PMC8931057 35301329

[40] Byrne, Á., Coombes, S. & Liddle, P. F. A neural mass model for abnormal beta-rebound in schizophrenia. 21–27 10.1007/978-3-030-18830-6_3 (2019).

[41] Arantes-Goncąlves, F., Wolman, A., Bastos-Leite, A. J. & Northoff, G. Scale for space and time experience in psychosis: converging phenomenological and psychopathological perspectives. *Psychopathology* **55**, 132–142 (2022). 10.1159/000519500 34872083

[42] Lechner, S. et al. From experience to symptoms: a multilayer hierarchy of psychopathological dimensions in schizophrenia. *Psychopathology* 10.1159/000547153/929646 (2025). 40587948

[43] Wengler, K., Goldberg, A. T., Chahine, G. & Horga, G. Distinct hierarchical alterations of intrinsic neural timescales account for different manifestations of psychosis. *eLife* **9**, 1–27 (2020). 10.7554/eLife.56151 PMC7591251 33107431

[44] Uscătescu, L. C. et al. Reduced intrinsic neural timescales in schizophrenia along posterior parietal and occipital areas. *npj Schizophr*. **7**, 55 (2021). 10.1038/s41537-021-00184-x PMC8608811 34811376

[45] Schaworonkow, N. & Nikulin, V. V. Spatial neuronal synchronization and the waveform of oscillations: implications for EEG and MEG. *PLoS Comput. Biol.* **15**, e1007055 (2019). 10.1371/journal.pcbi.1007055 PMC6534335 31086368

[46] Schaworonkow, N. & Nikulin, V. V. Is sensor space analysis good enough? Spatial patterns as a tool for assessing spatial mixing of EEG/MEG rhythms. *Neuroimage* **253**, 119093 (2022). 10.1016/j.neuroimage.2022.119093 35288283

