# Supplementary material for: Schizophrenia: temporal imprecision in phase, time, and frequency domains
Source: Schizophrenia (Heidelb). 2026 Aug 12;12(1):77. doi: 10.1038/s41537-026-00792-5 (PMC13614967; doi:10.1038/s41537-026-00792-5)
Supplement: Supplementary file 1 [file 41537_2026_792_MOESM1_ESM.docx]

**Supplementary material**

 **Supplementary Figure1: Correlations of neuronal measures with positive	and negative symptoms and medication dosage.** Only INT shows a nominal	significant correlation with positive symptoms that does not survive correction.

**Supplementary Figure2: Intra-subject dispersion of the FOOOF fit PSD and its components in the temporal lobe channels and all channels.** All channels aperiodic component p_Bonf = 0.0476, d = -0.532, all channels periodic component p_Bonf = 1.00, d = 0.191). Full FOOOF: p = 0.0139 d = -0.73, aperiodic: p = 0.0138 d = -0.785, periodic: p = 0.01 d = -0.093
